# Capital structure determinants across sectors: Comparison of observed evidences from the use of time series and panel data estimators

**DOI:** 10.1016/j.heliyon.2023.e19618

**Published:** 2023-09-07

**Authors:** Raja Rehan, Abdul Razak Abdul Hadi, Hafezali Iqbal Hussain, Qazi Muhammad Adnan Hye

**Affiliations:** a University Kuala Lumpur Business School, Kuala Lumpur, Malaysia; b Taylor's Business School, Taylor's University, Subang Jaya, Malaysia; c University of Economics and Human Sciences, Warsaw, Poland; d Academic Research and Development Wing, Dubai, United Arab Emirates

**Keywords:** Capital structure theories, Time series econometrics, Panel data analysis, Multiple regression, ARDL, GMM

## Abstract

This comparative study is an attempt to explore the determinants of capital structure for Malaysian firms listed in various sectors level. Within the framework of traditional and moderate dynamic capital structure theories, the key determinants such as fixed assets, current assets, return on equity, size, earning per share and total assets are tested in relation to the debt-equity ratio. The large-scale study entails data collected from 551 listed firms of Bursa Malaysia main market over 12 years period i.e. 2005-2016. Notably, this study combines Time Series econometrics with Panel Data analysis to enhance methodological robustness. Moreover, the comparative analysis approach is designated to recognize the most persistent capital structure determinants. In the first place, the Multiple Regression analysis (MRA) is selected as a baseline estimation method. Subsequently, the Auto Regression Distributed Lag model (ARDL), the Panel Data Static models, and Dynamic model via the Generalized Method of Moments (GMM) are employed to identify the capital structure determinants for the firms listed at Bursa Malaysia. The outcomes are surprising and indicate that the entire market is primarily controlled by the studied determinant total assets, which is significant in both construction and property sectors through MRA, ARDL, and GMM analysis. Technically, the significant role of tangibility and the existence of speed of adjustment across sectors imply that the Dynamic Capital Structure is the most prominent among all, followed by the Dynamic Trade-off theory.

## 1 Introduction

In the corporate finance literature, there have always been controversies and contradictory results among empirical inquiries when it comes to the topic of capital structure. Evidently, several studies have highlighted the importance of exploring the determinants that affect capital structure to boost an organization's financial performance. Capital Structure is referred to as the blend of debt and equity which is funded to a firm to carry out its operational activities and to attain strategic objectives of financial growth [1,2]. For this reason, the selection of debt-equity mix to formulate optimal capital structure and enhance profit margin to survive in the competitive environment is an important decision for the firms. Technically, an optimal capital structure is the best mix of debt and equity that raises the firm's market value and drops its overall cost of capital [3]. Despite myriad studies, the identification of determinants to articulate optimal capital structure is still unsettled. Nevertheless, the core capital structure theories such as Modigliani and Miller, Trade-Off, Pecking Order, and now their modern dynamic versions provide directions for the firms to select the most appropriate determinants that help them in formulating optimal capital structure [4]. Recently, academic investigations claim that the selection of best financing mix to formulate optimal capital structure can be done by combining various determinants that are associated with the firm's characteristics and its institutional-level settings [e.g. Refs. [5,6]]. Generally, a capital market is measured as an important institution of a country that delivers finance to improve the economic condition, performance, and industrial development [7]. Technically, sectors in the capital market are developed for those firms which have similar characteristics and nature of business [8].

Bursa Malaysia is one of the key capital markets and institutions that contribute meaningfully to Malaysian financial growth [9]. Earlier investigations that have been done in the context of Bursa Malaysia for investigating capital structure determinants deliver mixed and opposite results [see for e. g, Refs. [10,11]. In addition, with limited exceptions, previous investigations commonly do not offer any significant contribution, as they mainly focus on the firm's associated features instead of considering sector-specific determinants of capital structure [12,13]. Moreover, previous researchers have not provided a significant view and conclusive findings as their investigations chiefly focus on some specific sectors of the market, therefore, diverse and ambiguous outcomes are achieved [[14], [15], [16]]. Empirically, very limited studies have been conducted to explore the determinants of capital structure for Malaysian listed firms [17] and probe the implication of capital structure theories [18].

Moreover, prior inquiries suggest that the capital structure of Malaysian firms is not a static property and is dynamic in nature, hence, deviates from its targeted level [[19], [20], [21]]. Nevertheless, the presence of speed of adjustment (SOA) helps firms to return back toward their required targeted capital structure [22]. The SOA explicates the convergence period i.e. how speedily the firms move back to their required or targeted debt ratio [23]. A firm's targeted capital structure describes the capital structure which is optimal and a firm strives to maintain [24]. Unfortunately, in Malaysia, limited investigations have been conducted so far to recognize dynamic capital structure determinants [25,26]. Hence, the determinants of capital structure is still an unresolved issue in Malaysia and requires further broad investigation [27,28].

Motivated by the aforementioned facts, a comprehensive investigation considering the maximum sectors of Bursa Malaysia main market is warranted. Precisely, the sectors in Bursa Malaysia main market are categorized by dissimilar business models. Taking everything into account, this study is set to cover maximum sectors to explore the capital structure determinants for whole public listed firms at Bursa Malaysia. Therefore, in order to achieve these goals and to increase methodological robustness, this study combines Time Series econometrics with Panel Data analysis. Besides, this study also extends comparative analysis approach [e.g. [29, 30]] among the obtained findings from various econometrics techniques to come up with persistent capital structure determinants.

Beneficially, this study contributes to the existing literature and academia by delivering new empirical insights in several ways. Firstly, this study postulates a new knowledge of combining Time Series econometrics with Panel Data analysis that enhances the methodological robustness of this study. Evidently, the former studies delivered contradictory findings as they adopted dissimilar types of datasets and estimation approaches. Secondly, the comparative approach among various econometric techniques introduces a new way that also helps firms of other emerging markets to identify core sector-specific capital structure determinants. Thirdly, the use of a large data sample set of 551 individuals over 12 years i.e. 6612 observations by including firms from almost all sectors provides conclusive findings that would be beneficial for the whole Malaysian firms. Fourthly, the dynamic model is used to explore the point of optimal capital across all dissimilar sectors of Bursa Malaysia. This is to identify the trend of maintaining capital structure across the sectors and by listed Malaysian firms. Lastly, the observed outcomes are the baseline for policymakers and investigators to formulate a better financing model for Malaysian listed firms.

The rest of the study is structured as follows. Section 2 explains the literature review and theoretical framework. Section 3 exhibits the data sample construction and methodology. Subsequently, section 4 presents discussion for the empirical findings in details. After that, the conclusion part delivers a complete summary of the results with some recommendations for further future investigations.

## 2 Literature review & theoretical framework

### 2.1 Theoretical review of capital structure determinants

The present-day theories of capital structure are grounded on the effort of [31] who confirm that within the capital market that is considered perfect, the choice for selection of finance has no significant effects on the value of a firm. Afterwards, another theory which is named as Trade-Off theory postulates the idea of optimal capital structure and states that firms have chance to select their debt level which balances the financial cost disadvantages with tax benefits [32]. Later, Pecking Order theory is announced [33] which proposes the idea that firms initially emphasize more on internal funds then move for debt and then equity [34]. Technically, discussed capital structure theories are static in nature and explain capital structure related decisions of firms which belong to one term or period. This gives a way of entrance to new dynamic version of these theories [35]. announce a Dynamic Trade-Off theory which explains that a firm's capital structure cannot be at optimal stage all the time and it deviates from its optimal level which later requires necessary adjustment to return toward its optimal level. Similarly, the new model of Pecking-Order theory is presented by Ref. [36] that explains dynamic component and assumes that firms have flexibility of considering time element for their investment.

Next, the most relevant determinants that have been explained and reported significant by aforementioned capital structure theories in Malaysian context will be deliberated in details. Remarkably, this study proposes debt equity (DE) ratio as a dependent variable and total assets, fixed assets, current assets, size, return on equity and earnings per share as independent variables to measure capital structure. In addition, the empirical analysis of this study along with development of hypotheses is based on former studies. The earlier investigations enlighten variation in the selection of debt equity ratio at Bursa Malaysia [37]. This corroborates that capital structure ratio of firms in Malaysia is expected to be dynamic [38]. For that reason, it is obligatory to discover long-run, short-run and dynamic relationships among capital structure and its determinants. Remarkably, former inquiries specify that tangibility, sales (SIZE), liquidity, and profitability (ROE and EPS) elucidate capital structure of listed firms of Bursa Malaysia [see for e. g Refs. [39,40]]. Nevertheless, their association level is dissimilar i.e. positive association or negative association for these selected determinants. Thus, in view of prior inquiries, this study predicts positive relationships of liquidity, tangibility, SIZE and negative relationships for EPS and ROE with leverage. Hence, in order to find the answer of research question of this study that states: What are the core sector-specific capital structure determinants of the public listed firms at Bursa Malaysia? Following hypotheses are constructed:

H1 There is a long-run relationship between capital structure and determinants (Econometrics Time Series Analysis)

H2 There is a short-run relationship between capital structure and determinants (Econometrics Time Series Analysis)

H3 There is a significant relationship between capital structure and determinants (Dynamic Panel Data Analysis)

H4 There is a positive relationship between capital structure and liquidity (CA).

H5 There is a positive relationship between capital structure and tangibility (FA).

H6 There is a positive relationship between capital structure and tangibility (TA).

H7 There is a positive relationship between capital structure and SIZE.

H8 There is a negative relationship between capital structure and profitability (EPS).

H9 There is a negative relationship between capital structure and profitability (ROE%).

### 2.2 Empirical evidences of designated capital structure determinants

This section delivers the theoretical relevance and observed evidences for nominated dependent and independent variables of this empirical investigation.

### 2.3 Justification for using the dependent variable

#### 2.3.1 Debt equity ratio (DE)

Capital structure is the mixture of debt and equity that the firms acquire to finance their growth. The debt equity ratio is one of the major tested measures for capital structure and explained as total liabilities over total stockholders' equity [41]. Technically, this leverage ratio defines the relationship between the debt attained from creditors and acquired by shareholders to use as a firm's capital. The academic literature associated to capital structure explains a sequence of observed inquiries that use DE ratio as a measure for capital structure. For instance Ref. [42], in Malaysian context and [43] for Chinese firms employed debt equity ratio to explore firms' capital structure. Hence, as suggested by earlier studies [e.g. [44,45]] this study adopts debt equity ratio as a measure for capital structure.

### 2.4 Justification for using the independent variables

#### 2.4.1 Size

In this study, the size is measured as an independent variable represented by firm's annual sales figure. In context of Malaysia, earlier studies that investigate size as a capital structure determinant, report a positive relationship with a limited exception [e.g. Refs. [46,47]], however, it reveals mixed results in other contexts [e.g. [48,49]]. Notably, capital structure theories provide dissimilar views in term of relationship between sales and leverage. The Pecking Order theory forecasts a negative relationship between size and leverage of firms [50]. In contrast, the Trade-Off theory predicts a positive relationship and explains that in terms of profitability, the firms which are larger in size have minor chances of bankruptcy [51]. Following this, one may expect a positive relationship between the size of a firm and leverage.

#### 2.4.2 Return on equity (ROE)

ROE explains the efficiency of using shareholder's fund by a firm's management. The ratio of return on equity is defined as net income over total stockholders' funds [52]. Evidently, in various contexts, ROE as a capital structure determinant discloses diverse results for its relationship with capital structure. For example [53], in context of Malaysia, whereas [54] in context of Ghana exposes significant positive relationship of ROE with debt. Theoretically, the MM proposition states that projected return rate on equity rises proportionally with the debt-to-equity ratio [55]. In contrast, the Pecking Order theory explains that a firm selects internal finance first then debt and equity as the last alternative. Therefore, the Pecking Order theory proposes a negative relationship of profitability with the firm's capital structure [56]. Thus, in consistent with the earlier investigations, this study predicts negative relationship between ROE and capital structure.

#### 2.4.3 Earning per share (EPS)

EPS is a measure of profit that a firm has generated in a given period after tax, divided by the number of shares. Importantly, earlier literature specifies that EPS is a constantly positive and significant determinant of capital structure in Malaysian context with few exceptions [e.g. Refs. [57,58]]. According to the MM proposition II, share price of the leveraged firm is not affected and remains constant if increase in EPS is observed because of external leverage [59]. Clearly, this elucidates positive relationship between EPS and firm's capital structure. However, the Pecking Order theory favors to use retained earnings and clarifies negative relationship of EPS with capital structure [60]. Therefore, in view of Malaysian market where almost 80% firms are tagged as Shariah based firms which prefer to use internal finance first, this study accepts negative relationship of EPS with firms' capital structure.

#### 2.4.4 Current assets (CA)

This study introduces current assets as a capital structure determinant for listed Malaysian firms by using annual year-end figures of the firms' total current assets. Technically, current assets are liquid assets which offer extra figure of liquidity and help firms to easily access extra percentage of debt [61]. Likewise, to settle short term debt, a firm must have acceptable level of current assets. Notably, the motive to use this variable is that the major portion (80%) of the Malaysian capital market is covered by the Shariah firms [62], which prefer short term debt to avoid interest which is connected with the long-term debt financing. From academic literature [58], claim that Malaysian firms are able to meet short term creditors with their current assets. From theoretical explanation, the Pecking Order theory clarifies that a firm which owns excess current assets, preserves less debt ratio [63]. However, the Trade-Off theory deliberates current assets as a security measure to avail short term debt and offers a positive significant relationship between current assets and leverage. Having considered all things, this study predicts positive relationship between current asset and firms’ capital structure.

#### 2.4.5 Fixed assets (FA)

This study presents fixed assets as a key capital structure determinant of Malaysian firms by using end year stated figure of total fixed assets. Notably, the key purpose to deliberate fixed assets as a DE determinant is that the major fragment (80%) of Bursa Malaysia comprises of Shariah tagged firms that avoid interest-based debt and use its tangible assets as collateral to acquire more finance. Generally, Shariah-tagged firms are considered as tangible firms as they are not allowed to keep up with those assets which are receivable in nature, more than allowed percentage [64]. Similarly, in existence of sound fixed assets, stockholders consider their investment secure as they offer security to them against firm's bankruptcy [65]. Theoretically, the Trade-Off theory reflects a significant and positive relationship between firm's capital structure and its fixed assets as tangible assets are supportive to attain more debt from abundant sources. A few empirical inquiries support this claim, along with [66,67], who recognize positive and significant relationship between fixed assets and firms' leverage. Thus, this study predicts positive and significant relationship between fixed assets and capital structure.

#### 2.4.6 Total assets (TA)

Fundamentally, the most central determinant which impacts on the creation of capital structure is the firm's total assets [68]. Therefore, this study accepts total assets as a variable that is denoted by firms' reported end year total assets figures. Technically, assets of a firm consist of fixed and current assets and termed as total assets. From literature [69], in context of Iranian SMEs and [70] for Malaysian firms report total assets a significant determinant of leverage. Theoretically, the Pecking Order theory infers negative connection between firm's total assets and capital structure [71]. On the contrary, the Trade-Off theory describes that total assets are considered as a core security measure by lenders [72]. Therefore, the Trade-Off theory predicts a significant positive association between total assets and firms' capital structure. Hence, this study assumes positive significant relationship between total asset and firms' capital structure.

## 3 Data & samples construction

### 3.1 Sampling method and target population

The sample set of this study is constructed by engaging the Purposive Sampling method. Principally, Purposive Sampling is a process to generate a data sample in which the researcher depends on their own decisions while confirming targeted population to execute analysis [73,74]. Furthermore, this study uses quantitative approach to answer the nominated research questions. In order to perform the analysis, secondary data for a large-scale data sample set that is expressed in local Malaysian Ringgit (RM) is mined from the Bloomberg database. Moreover, the targeted population for this study is total 921 listed firms from 14 sectors (Trading & Services, Property, Infrastructure Project Companies (IPC), Industrial Products, Hotel, Consumer Products, Technology, Plantation, Construction, Finance, REIT, Mining, Special Purpose Acquisition Companies (SPAC), Close End Funds) of Bursa Malaysia main market.

### 3.2 Sample size and construction

In order to create final data sample set, Finance, REIT, Mining, Close End Funds and SPAC sectors are eliminated from the targeted population sample. Empirically, this is according to the practices of earlier studies [75]. The reason for not including the Finance and REIT sectors is that they are bound by local regulatory bodies to mitigate risk exposure, hence, their leverage-preserving practices are different [76]. Furthermore, limited data is accessible for three sectors i.e. Mining, SPAC and Close End Funds, thus, they are excluded from the sample. Next, after going through the filtering process, a large-scale data sample set of balanced Panel Data that comprises 551 listed firms over the 12-year period i.e. 2005-2016 is created. Technically, this provides total 6696 annual observations.

In the next step, the yearly average-based time series data sheet for selected nine sectors (Trading & Services, Property, IPC, Industrial Products, Hotel, Consumer Products, Technology, Plantation, and Construction) is created to perform econometrics time series analysis. Nevertheless, the assessment for dynamic capital structure determinants and speed of adjustment is subjected to survivorship bias as all those firms which are loss creating entities are omitted from the data sample set [77]. For that reason, data sample set size for dynamic assessment is reduced to 435 firms across seven sectors (Construction, Plantation, Property, Trading & Services, Technology, Consumer Products, and Industrial Products). Notably, SAS 9.4 analytical software is hired to perform empirical analysis on 551 firms’ data sample set.

## 4 Assessment methods and models specifications

Notably, this empirical study combines Time Series econometrics (Multiple Regression and ARDL) with Panel Data analysis (static models and dynamic model via GMM estimator) to boost the methodological robustness of the study. This is to recognize the key persistent determinants of capital structure across the selected sectors.

### 4.1 Multiple Regression analysis (baseline estimation method)

Multiple Regression (MR) is an econometrics technique that is used to explore the relationship between a sole dependent variable and multiple independent variables [78]. This study hires Multiple Regression estimation technique as a baseline method to perform comparative approach on 12-year average based time series data that is in line with the practices of former researchers in capital structure [see [79,80]]. Technically, a baseline method is used to generate forecasts for a created data sample set. By considering the assumptions of Multiple Regression, the projected theoretical model of this investigation is articulated which is explained in below equation (1):

(1) $${DE}_{t}=\alpha +\beta_{1}{TA}_{t}+\beta_{2}{SIZE}_{t}+\beta_{3}{ROE}_{t}+\beta_{4}\;{EPS}_{t}+\mu_{t}$$

where coefficients are represented by α and β, time specific effects are t, the error term is symbolised by μ_t_, Debt Equity ratio is represented by DE. Total assets is mentioned as TA, Sales is indicated by SIZE, Return on Equity is represented as ROE and finally Earning per Share is symbolised by EPS. Statistically, the Multiple Regression considers five basic assumptions which are linearity, expected zero value of error term, absence of multicollinearity, autocorrelation and heteroscedasticity [81]. However, this investigation performs variance inflation factor (VIF) test to detect multicollinearity issue, Durbin Watson D test for autocorrelation and White test to find heteroskedasticity problem in the model.

### 4.2 The Autoregressive Distributed Lag (ARDL) analysis

This study mobilized the Autoregressive Distributed Lag (ARDL) cointegration technique to capture short run and long run relationship between the selected variables. Fundamentally, cointegration technique is used as an econometrics method that constructs a model which has non-stationary variables and delivers meaningful results [82]. Remarkably, this investigation applies the exercise of ARDL cointegration technique by using 12-year average-based time series data which is consistent with the former researchers’ practices in capital structure [see Refs. [83,84]].

The main advantage of using the ARDL technique is that it can easily handle small data sample size [85]. Besides, ARDL permits to determine various lags of variables and accepts the relationship level either at level I (0) or I (1) or a mixture of both but not at I (2) [86]. Moreover, an Error Correction Model (ECM) of ARDL which is dynamic in nature can easily be formulated by a simple linear conversion. Technically, in ARDL estimation the pre-tests for unit root are not required [87]. Methodically, this study uses Schwarz Bayesian Criterion to determine the level of integration among the variables. Afterwards, the ARDL bound test is conducted to determine the long run relationship. When the long run relationship is established, this study moves to ARDL reparametrized Error Correction Model. The ARDL ECM model provides traditional ARDL results of long run relationship along with short run dynamic relationship of selected variables [88]. Hence, by following the work of former scholars [e.g. Ref. [89]] the ARDL long run estimation model of this investigation is presented in below equation (2):

(2) $${\text{DE}}_{t}=\alpha +\sum_{i=1}^{q}{\gamma_{1}}_{,j}\;{\left(\text{TA}\right)}_{t-i}+\sum_{i=0}^{p}{\gamma_{2}}_{,j}\;{\left(\text{SIZE}\right)}_{t-j}+\sum_{i=0}^{p}{\gamma_{3}}_{,j}\;{\left(\text{EPS}\right)}_{t-j}+\in_{t}$$

Here α is the coefficient and $\gamma_{2}$ to $\gamma_{6}$, j = 1, 2, …,3 signify the long run relationship of selected variables. Similarly, Debt Equity ratio is mentioned as DE, total assets by TA, sales by SIZE and earning per share by EPS. Next, after estimating the long run relationship, the ARDL model is reparametrized into the ECM model to find long and short run dynamic relationships. The articulated ECM model is presented in below equation (3):

(3) $$△{\text{DE}}_{t}=\alpha +\beta_{t}\left(\text{ECM}\right)+\sum_{i=1}^{q}{{Ϛ}_{1}}_{,j}△{\left(\text{TA}\right)}_{t-i}+\sum_{i=0}^{p}{{Ϛ}_{2}}_{,j}\;△{\left(\text{SIZE}\right)}_{t-j}+\sum_{i=0}^{p}{{Ϛ}_{3}}_{,j}\;△{\left(\text{EPS}\right)}_{t-j}+\in_{t}$$

Where α and $\beta_{t}$ are the estimated coefficient, ${Ϛ}_{1}$ to ${Ϛ}_{6}$, j = 1, 2, …,6 specify the short run dynamic relationship among the variables. Likewise, Debt Equity ratio is represented by △DE, total assets by △(TA), sales by △(SIZE) and earning per share by △(EPS). Statistically, the ARDL estimators assume no autocorrelation, absence of heteroskedasticity and normal distribution in the model [90]. Hence, this investigation conducts a Lagrange Multiplier (LM) test for serial correlation, Ramsey's RESET test for the model misspecification. Similarly, normality is checked by analyzing skewness and kurtosis of residuals and heteroscedasticity by analyzing regression of square residuals on squared fitted values.

### 4.3 Panel Data Static and Dynamic models

A Panel Data is a grouping of time-series and cross-sectional data [91]. The motives for this investigation to consider the balanced Panel Data Static and Dynamic modelling techniques are that the Panel Data enhances the degree of freedom. Besides, it also increases sample size, reduces diagnostic issues of multicollinearity, heteroscedasticity, heterogeneity and measures all those properties which are not acknowledged by cross-sectional data and time series data separately [92]. Analytically, this investigation starts with the condition of projected model which has been used by various prior researchers [e.g. Refs. [[93], [94], [95]]]. The constructed Panel Data model of this inquiry is displayed in below equation (4):

(4) $${\text{DE}}_{\text{it}}=\alpha_{i}{+\gamma_{t}+\beta }_{1}{\text{TA}}_{\text{it}}+\beta_{2}{\text{FA}}_{\text{it}}+\beta_{3}{\text{CA}}_{\text{it}}+\beta_{5}{\text{SIZE}}_{\text{it}}+\beta_{6}{\text{ROE}}_{\text{it}}+\beta_{7}{\text{EPS}}_{\text{it}}+\varepsilon_{\text{it}}$$

Here, i is occupied as individuals or firms (i = 1,2, ….551) and t is represented as time period of 12-year (t = 1,2, ….,12). Similarly, $\alpha_{i}$ specifies the cross sectional effect, $\gamma_{t}$ indicates the time series effect, $\beta_{1},\beta_{2,}\beta_{3},\beta_{4}$, $\beta_{5},\beta_{6}$ are the regression coefficients, DE designates debt to equity ratio, Total assets of firms is mentioned by TA, Fixed Assets by FA, Current Assets by CA, sales by SIZE, return on equity by ROE, earning per share by EPS and lastly $\varepsilon_{it}$ mentions error term effect. First, this investigation executes the Static Panel data analysis, followed by the Dynamic Panel data model via GMM estimator.

#### 4.3.1 Static Panel Data Models

The Panel Data Static Model is used to examine the individual behaviour or one-way effects in a repetitive environment [96]. The static panel models are further classified into three types: (i) Pooled Ordinary Least Squares (ii) Fixed Effects (iii) Random Effects. **Pooled Ordinary Least Square (POLS),** is the simplest model of the Panel Data analysis. The Ordinary Least Squares (OLS) is the basic and best procedure for evaluating the unspecified parameters in a regression analysis. Similarly, in **Fixed Effects model**, the individual and time series give its effects on intercept. In this model parameters are not random and have fixed quantities [97]. The constructed empirical model of the Fixed Effects investigation is displayed in below equation (5):

(5) $${\text{DE}}_{\text{it}}=\alpha +\beta_{1}{\text{TA}}_{\text{it}}+\beta_{2}{\text{FA}}_{\text{it}}+\beta_{3}{\text{CA}}_{\text{it}}+\beta_{5}{\text{SIZE}}_{\text{it}}+\beta_{6}{\text{ROE}}_{\text{it}}+\beta_{6}{\text{EPS}}_{\text{it}}++\gamma_{i}+\mu_{\text{it}}$$

Here, debt equity ratio is represented by DE. Likewise, time period t and individuals i followed by all other selected six explanatory variables i.e. TA, FA, CA, SIZE, EPS and ROE. Furthermore, α is an individual fixed parameter that is mentioned as a constant, β is taken as parameters matrix, $\gamma_{i}$ designates effect which is lying on intercept and $\mu_{it}$ mentions the error term. **In Random Effects model,** parameters are random variables and the time series and individuals leave its effects on slope [98]. Hence, the Random Effects model for this study is evaluated by using the model that is stated below in equation (6):

(6) $${\text{DE}}_{\text{it}}=\alpha +\beta_{1}{\text{TA}}_{\text{it}}+\beta_{2}{\text{FA}}_{\text{it}}+\beta_{3}{\text{CA}}_{\text{it}}+\beta_{4}{\text{SIZE}}_{\text{it}}+\beta_{5}{\text{EPS}}_{\text{it}}+\beta_{6}{\text{ROE}}_{\text{it}}+\mu_{i}+V_{t}+W_{\text{it}}$$

Here, DE is a dependent variable on individuals i and time period t which is followed by the selected six independent variables i.e. TA, FA, CA, SIZE, EPS and ROE. Likewise, β is considered as parameter matrix, $\mu_{i}$ is taken as an error term due to component which is cross section, $V_{t}$ is also mentioned as an error term which is because of time series, $W_{it}$ is error term because of time series and individual components. Statistically, to check the validity of the results attained from Panel data Static models' analysis, this investigation performs [99] Lagrange Multiplier (LM) test, Wallace and Hussain (VCR) test and Hausman test. Technically, the Lagrange Multiplier (LM) test is performed to check the selection for random or pooled effects model. Next, if the Random Effects model is found effective over the model of pooled OLS, then model is analyzed by Wallace and Hussain VCR component to approximate the Random Effects model. Lastly, the Hausman's test [100] is performed to compare and select the Random or Fixed Effects model for further investigations

#### 4.3.2 Dynamic Panel data model

Dynamic Panel model is used for investigations when current values of dependent variable depend on its past realization [101]. In other words, this model permits the use of dependent variable lags as an independent variable. Numerous empirical inquiries recommend that the firm's capital structure is not a static property and dynamic in nature [see [102,103]]. Notably, this study picks GMM estimator to examine Dynamic Panel model and SOA for the firms listed at various sectors of Bursa Malaysia. To check the robustness, this study engages difference GMM estimator that converts the independent variables by using a first difference, handle endogeneity and removes the unobserved fixed specific effect of the firm [104]. The dynamic effect model for GMM estimation of this study is specified in below equation (7):

(7) $${\text{DE}}_{\text{it}}=\beta_{1}\;{\text{DE}}_{i,t-1}+\beta_{2}{\text{TA}}_{\text{it}}+\beta_{3}{\text{FA}}_{\text{it}}+\beta_{4}{\text{CA}}_{\text{it}}+\beta_{5}{\text{SIZE}}_{\text{it}}+\beta_{6}{\text{ROE}}_{\text{it}}+\beta_{7}{\text{EPS}}_{\text{it}}+\mu_{\text{it}}$$

Here, ${DE}_{i,t-1}$ mentions lagged of dependen**t** variable i.e. ${DE}_{it}$. Likewise, by considering the difference GMM, the first modification in the dynamic model is given below in equation (8):

$$\Delta {\text{DE}}_{\text{it}}=\beta_{1}{\Delta DE}_{i,t-1}+\beta_{2}\Delta {\text{TA}}_{\text{it}}+\beta_{3}{\Delta FA}_{\text{it}}+\beta_{4}\Delta {\text{CA}}_{\text{it}}+\beta_{5}\Delta {\text{SIZE}}_{\text{it}}+\beta_{6}\Delta {\text{ROE}}_{\text{it}}+\beta_{7}\Delta {\text{EPS}}_{\text{it}}$$

(8) $$+\Delta \propto_{i}+\Delta \mu_{\text{it}}$$

By above equation (8), the dynamic model that is developed to estimate the speed of adjustment (SOA) is given below in equation (9):

(9) $${\text{DE}}_{\text{it}}=\left(1-\lambda \right)\;{\text{DE}}_{it-1}+\beta_{1}{\text{TA}}_{\text{it}}+\beta_{2}{\text{FA}}_{\text{it}}+\beta_{3}{\text{CA}}_{\text{it}}+\beta_{4}{\text{SIZE}}_{\text{it}}+\beta_{5}{\text{ROE}}_{\text{it}}+\beta_{6}{\text{EPS}}_{\text{it}}+\mu_{\text{it}}$$

Here, variables of equation (9) are well-defined in above equation (see equation (4)). Debt Equity ratio is denoted by ${DE}_{it}$ and, $\left(1-\lambda \right)\;{DE}_{it-1}$ represents lagged value of dependent variable that is used for evaluating speed of adjustment for a firm to sustain targeted capital structure. Moreover, this study estimates SOA by deducting coefficient value ‘λ’ from 1 i.e. (1-λ) which specifies the alteration between targeted level of debt and actual level of debt [105]. Statistically, to handle the diagnostic issue for the GMM analysis, this study selects Sargan test to check the exogeneity issue. Moreover, Autocorrelation AR (m) test is used to check serial correlation issue in the models [106].

## 5 Empirical findings and discussions

This section delivers a comprehensive discussion on the observed findings. Prior to the Multiple Regression and ARDL estimations, the mentioned diagnostic tests (see sec 4.1, 4.2) are executed (see Appendix A) to determine the goodness of fit of the sectors models. In spite of some heteroscedasticity and multicollinearity issues, the outcomes settle that the models are effective in most of the cases and show satisfactory goodness of fit (see Appendix A). Notably, to get significant results, the numbers of independent variables have been reduced to four (TA, SIZE, ROE, EPS) in Multiple Regression and ARDL analysis due to the presence of multicollinearity between total assets and its subcomponents namely fixed and current assets. Later, another variable i.e. ROE is eliminated from the ARDL model as it contains large number of negative observations. Analytically, this is according to the practices of former studies and researches [see [107,108]]. Similarly, for GMM estimation all the mentioned diagnostics tests (see sub sec 4, 4.3.1) confirm that models are correctly specified and have the greater potential to predict (see Appendix A).

Moreover, related theories are deliberated carefully and the strength of each supportive theory is emphasized. Likewise, the consistency level between the findings of the baseline estimation method i.e. Multiple Regression with ARDL and then with GMM results are also accurately cross-examined. To start up, the descriptive statistics of 551 public listed Malaysian firms over the 12-year period (2005–2016) are presented in Table 1 below:

**Table 1:** Descriptive Statistics (551 Public listed Malaysian Firms).

Variables	Maximum	Minimum	Mean	Median	Mode	Std. Dev
DE	37.85200	0.0039175	0.8931	0.614261	0.050461	1.099172
TA	132,902.2	3.785	1617.08	311.4633	529.457	6581.65
FA	101,685.4	0.2749	715.4098	98.1873	129.0761	3881.64
CA	81,459.81	2.7954	586.5266	147.532	9.8533	2223.91
SIZE	47,254.5	2.415	830.4939	200.4752	10.28644	2759.23
ROE	148.3993	−9.283	0.111308	0.0675916	0.162262	2.369519
EPS	72.3870	−9.243	0.128427	0.0536	0.020000	1.120352

Note: DE = Debt Equity Ratio, TA = Total Assets, FA= Fixed Assets, CA =Current Assets, SIZE = Sales, ROE = Return on Equity, EPS = Earnings Per Share.

Looking at statistical results obtained from descriptive analysis, it is clear that mean DE is reported at 0.8931% and join with the median of 61% which is moderately low. Likewise, the maximum TA is recorded at 1.32 billion but its mode stands at minor level of 529 million. The mean FA and CA are registered at 715 million and 586 million respectively. On the back of high mean value of sales revenue of RM830 million, the percentage mean of ROE is reported around 11.1. Interestingly, the EPS mean also stands 12.8% that is high, delivering solid earning to the listed firms’ stockholders. Lastly, from the descriptive analysis results for standard deviation, it seems that there has been a large degree of dispersions among all the studied variables. Notably, the preliminary results look credible and provide support to the above discussed capital structure theories discussed in the literature. Next, Table 2 presents the empirical findings obtained from Multiple Regression analysis. Analytically, this study uses Multiple Regression as a base line estimation method and for a comparative approach to identify core capital structure determinants of firms at Bursa Malaysia.

**Table 2:** Empirical findings from multiple regression (MR) analysis.

S #	Sectors	TA		SIZE		ROE		EPS	
		Estimate	p-value	Estimate	p-value	Estimate	p-value	Estimate	p-value
1	Prop.	−0.0018	0.0021**	0.008	0.0019**	0.0691	0.0175**	−2.588	0.0029**
2	Const.	0.0004	0.0136**	−0.0015	0.0261**	6.1525	0.0408**	−6.057	0.0478*
3	IPC	−0.0008	0.1979	0.0003	0.2848	2.8505	0.0596*	−2.1421	0.3581
4	Ind. Prod.	−0.0000	0.9492	−0.0004	0.1329	4.3613	0.0532*	−0.4938	0.1958
5	Trad/Ser	−0.0000	0.5987	−0.0002	0.0047**	0.0746	0.2987	−0.0514	0.3152
6	Hotel	−0.0017	0.0161**	−0.0008	0.5958	−7.1247	0.1631	1.6737	0.3225
7	Con. Prod.	−0.0001	0.6738	−0.0002	0.7232	−0.0650	0.3070	−0.0289	0.9682
8	Tech	−0.0004	0.8528	0.0005	0.8443	−1.7185	0.3479	−0.6342	0.7055
9	Plant.	−0.0000	0.9324	0.0000	0.5130	−0.0629	0.9378	0.0683	0.5882

***, **, * Significant at 1%, 5% and 10% level Note: TA = Total Assets, Size = Sales, ROE = Return on Equity, EPS = Earnings Per Share, Prop. = Property, Const. = Construction, Con. Prod. = Consumer Products, Ind. Prod. = Industrial Products, Tech. = Technology, Trad/Ser = Trading & Services, Plant. = Plantation.

Subsequently, the ARDL bound testing approach is executed to find long run relations among studied variable. Technically, the null hypothesis of no cointegration (H_0_: A long run relationship does not exist) is rejected if F-Statistics outcome is greater than (F > Upper Bound) the value of its estimated upper bound. Moreover, the negative ECM coefficient (less than −1 or between 0 and -1) of ARDL ECM model and its significant p-value are used to describe the long-run and short-run dynamic relationship among the variables [109]. The following table summarizes the results obtained from the ARDL bound testing and long run coefficients estimation.

Table 3 above presents the findings obtained from ARDL bound testing technique. First, this study selects lag order by using Schwarz-Bayesian Criterion as long run coefficients calculation is very sensitive to its lag length order selection [110]. After that, the ARDL bound testing approach is applied. Evidently, the F-Statistics outcomes for Industrial Products and Hotel sectors are greater than its upper bound critical values. Statistically, this specifies the presence of a long run relationship between capital structure and its determinants in Industrial Products (F > 8.284) and Hotel (F > 6.5903) sectors. Hence, hypothesis H_1_ of long-run relationship is accepted for these two sectors. Furthermore, the ARDL Normalized regression result mentions the significant capital structure determinants for investigated sectors. Evidently, the positive and significant long-run relationship of TA in Hotel and Industrial Products sectors indicate that these sectors efficiently maintain tangibility and are capable of using more leverage. The significant tangibility and sales directly suggest the application of Trade-Off theory in describing firms’ choices of capital structure in these sectors. The empirical findings from the Hotel sector are consistent with the study of [111] who conclude that asset tangibility is more influential determinant of Portuguese Hotel sector firms. Similarly, the empirical findings from the Industrial Products sector are in line with the results of [112] which describe significant tangibility for the listed Malaysian firms across various sectors including the Industrial Products sector.

**Table 3:** (a): ARDL bound-testing approach and long run coefficients models ARDL bound tests for the existence of a long run relationship among the variables. (b) ARDL Normalized Regression Results Based on Schwarz Bayesian Criterion.

H_0_: A long run relationship does not exist. H_1_: A long run relationship exists.						
S#	Sectors	F-Statistic > Upper Bound	95% Lower Bound	95% Upper Bound	90% Lower Bound	90% Upper Bound
1	Prop.	0.8217	3.992	5.961	2.906	4.39
2	Const.	3.029	4.284	6.59	3.029	4.736
3	IPC	3.375	3.992	5.961	2.906	4.39
4	Ind. Prod.	8.284	4.285	6.59	3.029	4.736
5	Trad/Ser	0.952	3.992	5.961	2.906	4.39
6	Hotel	491.702	4.285	6.59	3.028	4.736
7	Con. Prod.	1.414	4.284	6.59	3.029	4.736
8	Tech	3.828	4.284	6.59	3.029	4.736
9	Plant.	0.634	3.992	5.961	2.906	4.39

(b)							
S#	Sectors	TA		SIZE		EPS	
		Estimate	p-value	Estimate	p-value	Estimate	p-value
1	Prop. (0,0,0,0,1)	−0.0013	0.034**	0.0067	0.010**	−1.7958	0.063*
2	Const. (1,1,0,1,1)	0.0011	0.057**	−0.0028	0.136	2.0223	0.578
3	IPC (0,1,0,0,1)	0.0026	0.175	0.0017	0.028**	−7.6038	0.106
4	Ind. Prod. (1,0,1,1,1)]	0.1201	0.061*	−0.1255	0.139	0.0294	0.873
5	Trad/Ser (0,0,0,0,0)	0.75502	0.003**	−6.6995	0.957	1.3245	0.000***
6	Hotel (1,1,1,1,1)	−0.0018	0.059**	−0.0363	0.097*	11.0046	0.090*
7	Con.Prod. (1,1,1,0,1)]	0.0013	0.012**	−0.8108	0.184	3.5953	0.023**
8	Tech (1,1,0,1,1)	−0.0026	0.392	−0.0015	0.677	0.5163	0.658
9	Plant. (0,0,0,0,1)	0.1026	0.959	0.9528	0.612	0.0596	0.593

***, **, * Significant at 1%, 5% and 10% level Note: TA = Total Assets, SIZE = Sales, EPS = Earnings Per Share, Prop. = Property, Const. = Construction, Con. Prod. = Consumer Products, Ind. Prod. = Industrial Products, Tech. = Technology, Trad/Ser = Trading & Services, Plant. = Plantation.

After establishing the long-run relationship, the ARDL model is reparametrized into ECM model to inspect long and short run dynamic relationships. The lag order for the ECM model is selected on the basis of Schwartz Bayesian Criteria. Clearly, the obtained results in above Table 4 indicates the establishment of short-run relationship between leverage and studied capital structure determinants of Trading & Services (TA, EPS), IPC (SIZE), Industrial Products (SIZE), Plantation (EPS), Hotel (TA, EPS, SIZE) and Consumer Products (TA, EPS, SIZE) sectors. Considering the statistical results obtained from ARDL ECM approach, the hypothesis H_2_ of significant short-run relationship is accepted for these sectors. Evidently, the ECM model of Consumer Products sector specifies exciting results, whereby, the ECM coefficient is equal to −0.7776 for DE in the short run which infers that deviation from the long run equilibrium is corrected by 77.76% over every year. The significant role of tangibility and sales specifies the importance of Trade-Off theory for the Malaysian market. The findings are in line with the results of [113] who postulate long and short run relationship of tangibility and sales with leverage in various sectors of Pakistani capital market and conclude that sector level setting affects on firms’ capital selection choices. The following Table 5 portrays a comparative analysis of findings attained from time series techniques i.e. MR and ARDL across sectors. The literature review section provides the consistency of findings with the results of earlier investigations.

**Table 4:** ARDL error correction model (ECM) based on Schwarz Bayesian criterion.

S#	Sectors	TA		SIZE		EPS		ecm (−1)	
		Estimate	p-value	Estimate	p-value	Estimate	p-value	Estimate	p-value
1	Prop. (1,1,1,1,1)	−0.76951	0.503	0.002998	0.298	−0.76951	0.503	−0.0195	0.856
2	Const. (1,1,0,1,1)	0.7427	0.128	−0.9869	0.154	−0.0737	0.952	−0.6213	0.910
3	IPC (0,1,0,0,1)	−0.375	0.838	0.0011	0.008*	−0.4435	0.822	−1.568	0.005**
4	Ind. Prod. (1,0,1,1,1)	−0.294	0.980	−0.475	0.024**	0.067	0.870	−2.296	0.004**
5	Trad/Ser (0,0,0,0,0)	0.874	0.029**	111.8	0.549	1.128	0.003**	−0.001	0.990
6	Hotel (1,1,1,1,1)	0.001	0.000***	0.003	0.000***	−1.264	0.000***	0.172	0.030**
7	Con. Prod. (1,0,0,1,1)	0.001	0.001**	−0.863	0.017**	1.022	0.038**	−0.777	0.000**
8	Tech (1,1,0,1,1)	−0.004	0.4120	0.003	0.425	−1.784	0.205	−1.583	0.007*
9	Plant. (0,0,0,0,1)	0.925	0.9350	−0.639	0.976	0.226	0.080*	−0.146	0.326

***, **, * Significant at 1%, 5% and 10% level Note: TA = Total Assets, SIZE = Sales, EPS = Earnings Per Share, Prop. = Property, Const. = Construction, Con. Prod. = Consumer Products, Ind. Prod. = Industrial Products, Tech. = Technology, Trad/Ser = Trading & Services, Plant. = Plantation.

**Table 5:** Time series analysis for capital structure determinants across sectors.

S#	Sectors	Significant Variables		MR vs. ARDL	Literature Review		
		Multiple Regression (MR) Results (p-values)	ARDL Normalized Regression Results (p-values)		Authors	Subject/Period	Findings
1	Prop.	-TA (0.0021**), SIZE (0.0019**), ROE (0.0175**), -EPS (0.0029**)	-TA (0.034**), SIZE (0.010* *), -EPS (0.063*)	MR = ARDL	Wahab, Amin and Yusop (2012)	Malaysia (2001–2010)	Significant Tangibility & Profitability
2	Const.	TA (0.0136**), -SIZE (0.0261**), ROE (0.0408**), -EPS (0.0478**)	TA (0.057**)	MR = ARDL	Ahmad and Azhar, (2015)	Malaysia (2009–2013)	Significant Tangibility
3	IPC	ROE (0.0596**)	SIZE (0.028**)	MR ≠ ARDL	Gwatidzo and Ojah (2012)	South Africa (1990–2005)	Significant SIZE
4	Ind. Prod.	ROE (0.0532**)	TA (0.061*)	MR ≠ ARDL	Sahudin et al. (2019)	Malaysia (2002–2011)	Significant Tangibility
5	Trad/Ser	-SIZE (0.0047**)	TA (0.003**), EPS (0.000***)	MR ≠ ARDL	Baharuddin et al. (2011)	Malaysia (2001–2007)	Significant Tangibility
6	Hotel	-TA (0.00161**)	-TA (0.059**), -SIZE (0.097*), EPS (0.090*)	MR ≠ ARDL	Matias, Salsa and Afonso (2018)	Portuguese (2006–2014)	Significant Tangibility
7	Con. Prod.	–	TA (0.012**), EPS (0.023**)	MR ≠ ARDL	Noraidi and Ramakrishna (2018)	Malaysia (2006–2015)	Significant Tangibility
8	Plant.	–	–	MR = ARDL	Ramakrishnan (2012)	Malaysia (1996–2007)	Insignificant Profitability
9	Tech	–	–	MR = ARDL	Ramakrishnan (2012)	Malaysia (1996–2007)	Insignificant Profitability

***, **, * Significant at 1%, 5% and 10% level Note: TA = Total Assets, SIZE = Sales, EPS = Earnings Per Share, ROE = Return on Equity, Prop. = Property, Const. = Construction, Con. Prod. = Consumer Products, Ind. Prod. = Industrial Products, Tech. = Technology, Trad/Ser = Trading & Services, Plant. = Plantation, MR = Multiple Regression, ARDL = Autoregressive Distributed Lag.

Evidently, the comparative analysis in above table mentions that at sectors level, the selection for debt equity choices are dissimilar because of each sector's internal settings. Clearly, the outcomes obtained from both time series estimators which are MR and ARDL indicate that each sector has dissimilar significant capital structure determinants. However, the consistency level between MR and ARDL findings is detected for Plantation, Property, Technology and Construction sectors. Clearly, the comparative approach elucidates that Malaysian market is mainly controlled and measured by studied determinants total assets (TA), with the exclusion of Plantation, Technology and IPC sectors. Remarkably, the former investigations indicate the same point that asset tangibility is a key factor that defines Malaysian firms' capital structure choices [e.g. Refs. [[114], [115], [116]]]. The utmost significant TA at numerous sectors of Bursa Malaysia elucidates that Trade-Off theory is more supportive to describe capital structure choices of the firms at Bursa Malaysia.

Additionally, this study also endeavors to deliver an in-depth knowledge on the dynamic relationship for the sector-specific determinants with capital structure. In order to get robust results at this level, this study explores dynamic capital structure determinants via GMM estimator. Notably, the GMM analysis is subject to survivorship bias. The following table elucidates the comparative evaluation between the findings of the GMM analysis and the Multiple Regression techniques for the enduring sectors of Bursa Malaysia.

Table 6 above reveals the conclusions attained from the GMM estimation and the Multiple Regression (MR) technique which deliver inconsistency among all the sectors with the exemption of the Construction sector. The results expose that asset tangibility is a main determinant which controls the whole Bursa Malaysia. Similarly, the Dynamic Capital Structure determinants vary across the investigated sectors. Technically, the presence of dynamic capital structure designates the occurrence of targeted capital structure and speed of adjustment for the listed Malaysian firms at sectors level. Therefore, the following Table 7 displays the lagged dependent variable coefficients across the seven sectors that are found to be statistically significant and SOA for each selected sector.

**Table 6:** Panel data analysis on capital structure determinants across sectors.

S#	Sectors	Significant Variables Results		MR vs. GMM	Literature Review		
					Authors	Period/Subject	Findings
		Multiple Regression (p-values)	GMM Estimator (p-values)				
1	Const.	TA (0.0136**), -SIZE (0.0261**), ROE (0.0408**), -EPS(0.0478**)	TA (<.0004***), FA (0.0051**), CA (0.0002***), -SIZE (0.0005**)	MR = GMM	Baharuddin et al. (2011)	Malaysia (2001–2007)	Significant Tangibility
2	Con. Prod.	–	FA (<.0001***), CA (<.0001***)	MR ≠ GMM	Noraidi And Ramakrishna (2018)	Malaysia (2006–2015)	Significant Tangibility
3	Ind. Prod	ROE (0.0532**)	TA (0.00210**), FA (<.0001***), ROE (0.009*), -EPS(0.0320**)	MR ≠ GMM	Sahudin et al. (2019)	Malaysia (2002–2011)	Significant Tangibility
4	Plant.	–	TA (0.0170**), CA (<.0001***), -EPS(0.0211**)	MR ≠ GMM	Sahudin et al. (2019)	Malaysia (2002–2011)	Significant Tangibility, Liquidity & Profitability
5	Prop.	-TA (0.0021**), SIZE (0.0019**), ROE (0.0175**), -EPS (0.0029**)	FA (0.0009**)	MR ≠ GMM	Wahab, Amin and Yusop (2012)	Malaysia (2001–2010)	Significant Tangibility & Profitability
6	Tech	–	CA (0.0054**)	MR ≠ GMM	Hussain and Miras (2015)	Malaysia (1997–2011)	Significant Liquidity
7	Trad/Ser	-SIZE (0.0047**)	TA (<.0001***), -FA (0.0009***), ROE (0.0053**)	MR ≠ GMM	Sahudin et al. (2019)	Malaysia (2002–2011)	Significant Tangibility& Profitability

***, **, * Significant at 1%, 5% and 10% level Note: TA = Total Assets, SIZE = Sales, EPS = Earnings Per Share, ROE = Return on Equity, Prop. = Property, Const. = Construction, Con. Prod. = Consumer Products, Ind. Prod. = Industrial Products, Tech. = Technology, Trad/Ser = Trading & Services, Plant. = Plantation, MR = Multiple Regression, GMM = the Generalized Method of Moments.

**Table 7:** Dynamic GMM analysis and the speed of adjustment (SOA) across sectors.

S#	Sector	Coeff.	*P*-Value	SOA	Authors	Subject	Findings
1	Ind. Prod.	0.1978	0.0118**	80.20%	Gwatidzo and Ojah (2012)	South Africa	Rapid SOA of a firm is a function of its determinants growth, size and tangibility of assets.
2	Con. Prod.	0.202	0.0062*	79.80%	Haron and Ibrahim (2012)	Malaysia	High and rapid SOA for Malaysian listed firms.
3	Prop.	0.2412	0.0082*	75.80%	Halim, Sukor and Bacha (2019)	Malaysia	High and rapid SOA for all various level of Malaysian Shariah firms.
4	Trad/Ser	0.2547	0.0017**	74.00%	Mukherjee and Mahakud (2010)	India	Firms' target debt level is a function of its asset tangibility, size of firm and profitability.
5	Tech	0.3835	0.0523**	61.60%	Haron and Ibrahim (2012)	Malaysia	High SOA for Malaysian listed firms.
6	Const.	0.397	<.0001***	60.30%	Marsh (1982)	United Kingdom	Firms' target debt level is a function of its asset composition and size
7	Plant.	0.5794	<.0001***	42.00%	Abdeljawad et al. (2013)	Malaysia	Slow SOA for the Malaysian listed firms.

***, **, * Significant at 1%, 5% and 10% level.

Note: Coeff. = Coefficient, Const. = Construction, Con. Prod. = Consumer Products, Ind. Prod. = Industrial Products, Tech. = Technology, Trad/Ser = Trading & Services, Prop. = Property, Plant. = Plantation, SOA= Speed of Adjustment.

Clearly, the evidence in above table provisions the presence of targeted capital structure as claimed by Ref. [117] clarifies the effects of dynamic forces behind the theories of capital structure. This study discovers that the SOA travels at a rapid pace across the selected sectors, from minimum of 42.0% up to the maximum of 80.2%. Under this examination of Dynamic Target Capital Structure, an average listed firm from these sectors progressively travels toward its suboptimal capital structure to the optimal level of 67.67% per year. Needless to say, significant lagged dependent variables and the presence of speed of adjustment across these sectors confirm that Dynamic Capital Structure and SOA do exist in the firms listed at Bursa Malaysia. Thus, the hypothesis H_3_ for significant dynamic relationship is accepted for all of the seven sectors. Analytically, the results from GMM analysis specify that Dynamic or Targeted Capital Structure is the most prominent amongst all, shadowed by the Dynamic Trade-Off Theory. The findings are in line with the former studies of [[118], [119], [120]] that confirm the presence of dynamic or targeted capital structure and speed of adjustment.

As a whole, this investigation recognizes that the determinants choices of capital structure are not similar at various sectors levels because of each sector's internal and dissimilar settings. Therefore, in view of the maximum findings that are obtained by MR, ARDL and GMM estimators, the hypotheses H_4,_ H_5,_ H_6,_ and H_7_ for the positive relationships among CA, FA, CA, SIZE and capital structure are accepted. Likewise, hypotheses H_8_ and H_9_ are accepted for EPS and ROE that postulate negative relationship among capital structure studied determinants. The findings are consistent with the earlier studies of [121,122] which conclude that sector or industrial setting affects firms' level capital structure determinants choices. At the onset, this study lays emphasis on key competing theories, however, the Dynamic Trade-Off Capital Structure theory emerges as the most prominent among all the theories, across investigated sectors at Bursa Malaysia.

## 6 Conclusion & limitations

This empirical investigation is comprehensive indeed as it examines sector-specific determinants of capital structure for 551 public listed firms at Bursa Malaysia over the period of 2005–2016. The key traditional capital structure theories which include Modigliani-Miller theory, Trade-Off theory, Pecking Order theory and modern Dynamic Capital Structure theories are put into test in this investigation. The results provide strong evidence that the choices for capital structure determinants are different across sectors, pointing out the importance of sector level analysis and despite the fact that the market is mainly controlled by determinant total assets. Even though the financing requirements and patterns in each individual sector are different, firms’ practice to maintain asset tangibility seem to be almost similar which is significant in both construction and property sectors across MRA, ARDL and GMM analysis. One possible reason behind the significance of this determinant is that maximum market fragment (80%) is covered by the Shariah firms which are generally considered as tangible firms [123]. The debt facilities awarded to Shariah firms are asset-backed and offered after considering their assets tangibility [124]. As a whole, the significant tangibility and lagged dependent variables across the sectors directly specify the relevance of Dynamic Trade-Off theory which describes capital structure preserving practices of listed firms at Bursa Malaysia.

The core limitation of this study is the ARDL and the Multiple Regression-based study data structure. Several data points are removed due to multicollinearity, serial correlation and heteroscedasticity issues. Thus, the use of only 13 data points over the years creates a constraint on degree of freedom and parameter estimates. Also, it is important to note that Bursa Malaysia recategorized its sectors classification in September 2018. Therefore, future researchers should add more data point while investigating capital structure determinants for Malaysian-listed firms. Several steps could be taken to reduce diagnostic issues of the models and this includes an increase in sample size and an elimination of highly correlated variables. Likewise, future researchers should consider new sectors classification in Bursa Malaysia main market.

## Author contribution statement

Raja Rehan: Conceived and designed the experiments; Performed the experiments; Wrote the paper.

Abdul Razak Abdul Hadi: Performed the experiments; Analyzed and interpreted the data.

Hafezali Iqbal Hussain: Qazi Muhammad Adnan Hye: Contributed reagents, materials, analysis tools or data.

## Data availability statement

Data will be made available on request.

## Additional information

No additional information is available for this paper.

## Declaration of competing interest

The authors declare that they have no known competing financial interests or personal relationships that could have appeared to influence the work reported in this paper.

## Figures and Tables

**Table 1a:** Multiple Regression Diagnostic Tests for Sector-Specific Models

Sectors									
Variables	Trading & Services			Property			Industrial Products		
	VIF	DW D (*P*-value)	Moment Spec. H_0_ (*P*-value)	VIF	DW D (*P*-value)	Moment Spec. H_0_ (*P*-value)	VIF	DW D (*P*-value)	Moment Spec. H_0_ (*P*-value)
**TA**	6.955	0.718	0.6138	122.414	0.8484	0.5778	1.57473	0.4872	0.4086
**SIZE**	6.182			148.339			1.45099		
**ROE**	1.581			1.14193			1.850554		
**EPS**	1.153			4.63173			1.48351		
**Variables**	**Plantation**			**IPC**			**Construction**		
	**VIF**	**DW D (*P*-value)**	**Moment Spec. H**_**0**_**(*P*-value)**	**VIF**	**DW D (*P*-value)**	**Moment Spec. H**_**0**_**(*P*-value)**	**VIF**	**DW D (*P*-value)**	**Moment Spec. H**_**0**_**(*P*-value)**
**TA**	13.493	0.139	0.732	2.634	0.469	0.885	3.67821	0.0929	0.3928
**SIZE**	11.186			3.477			3.65756		
**ROE**	3.185			4.56			4.84118		
**EPS**	2.134			3.343			6.46248		
**Variables**	**Hotel**			**Consumer Products**			**Technology**		
	**VIF**	**DW D (*P*-value)**	**Moment Spec. H**_**0**_**(*P*-value)**	**VIF**	**DW D (*P*-value)**	**Moment Spec. H**_**0**_**(*P*-value)**	**VIF**	**DW D (*P*-value)**	**Moment Spec. H**_**0**_**(*P*-value)**
**TA**	22.106	0.1815	0.4772	18.40653	0.1605	0.7693	8.05508	0.219	0.5744
**SIZE**	1.47966			18.96982			12.4181		
**ROE**	130.015			2.73152			8.57307		
**EPS**	72.215			4.00629			2.86468		

Note: VIF = Variance Inflation Factor, DW D = Durbin Watson D.

**Table 2a:** ARDL Diagnostic Tests for Sector-Specific Models

Diagnostic Tests	Trad/Ser	Prop.	IPC	Ind. Prod.	Hotel	Con. Prod.	Tech.	Plant.	Const,
	F-Statistic	F-Statistic	F-Statistic	F-Statistic	F-Statistic	F-Statistic	F-Statistic	F-Statistic	F-Statistic
	(*P*-value)	(*P*-value)	(*P*-value)	(*P*-value)	(*P*-value)	(*P*-value)	(*P*-value)	(*P*-value)	(*P*-value)
**A: Serial Correlation**	0.37887 (0.561) >α (0.05)	2.86621 (0.151) > α (0.05)	.12,122 (0.745) > α (0.05)	1.1518 (0.395) > α (0.05)	None	100.6566 (0.010) > α (0.05)	6.2430 (0.130) > α (0.05)	0.00228 (0.964) > α (0.05)	10.9676 (0.080) > α (0.05)
**B: Functional Form**	1.7505 (0.234) > α (0.05)	0.56562 (0.486) > α (0.05)	4.9934 (0.089) > α (0.05)	None	None	None	Not applicable	0.0099723 (0.924) > α (0.05)	None
**D: Heteroscedasticity**	0.04029 (0.845) > α (0.05)	1.1179 (0.318) > α (0.05)	7.8521 (0.021) > α (0.05)	37,341 (0.556) > α (0.05)	0.75023 (0.409) > α (0.05)	0.10930 (0.749) > α (0.05)	5.23301 (0.048) > α (0.05)	0.89769 (0.368) > α (0.05)	1.4602 (0.258) > α (0.05)

Note: Prop. = Property, Const. = Construction, Con. Prod. = Consumer Products, Ind. Prod. = Industrial Products, Tech. = Technology, Trad/Ser = Trading & Services, Plant. = Plantation.

**Table 3a:** Sargan Test results of GMM for Sector-Specific Models

Sargan Test for Exogeneity of Instruments H_0_: The instruments used in this model are valid instruments H_1_: The instruments used in this model are not valid instruments							
**Sectors**	**Construction**	**Consumer Products**	**Industrial Products**	**Plantation**	**Property**	**Technology**	**Trading & Services**
(Prob > ChiSq)	0.7309	0.5434	0.7868	0.592	0.6986	0.9998	0.7802
Number of cross sections	27	80	127	36	46	17	102

**Table 4a:** Auto Correlation AR (m) Test results of GMM for Sector-Specific Model

AR (m) test on Error Terms H_0_: There is no auto correlation on error terms H_1_: Auto correlation is present on error terms							
**Sectors**	**Construction**	**Consumer Products**	**Industrial Products**	**Plantation**	**Property**	**Technology**	**Trading & Services**
(Prob > Statistic)	0.6647	0.9955	0.9998	0.9941	0.871	0.2283	0.9855
Number of cross sections	27	80	127	36	46	17	102
